# Assessing the Biocompatibility of Tannic Acid-Based Biomaterials: Addressing Challenges in Standard Cytotoxic Assays

**DOI:** 10.3390/bioengineering12060660

**Published:** 2025-06-16

**Authors:** Silvia Cometta, Dietmar Werner Hutmacher

**Affiliations:** 1 Faculty of Engineering, School of Mechanical, Medical and Process Engineering, Queensland University of Technology, Brisbane, QLD 4000, Australia; 2 Australian Research Council Training Centre for Multiscale 3D Imaging, Modelling and Manufacturing (M3D Innovation), Queensland University of Technology, Kelvin Grove, QLD 4059, Australia; 3 Max Planck Queensland Centre, Queensland University of Technology, Brisbane, QLD 4000, Australia; 4 Australian Research Council Training Centre for Cell and Tissue Engineering Technologies, Queensland University of Technology, Brisbane, QLD 4059, Australia

**Keywords:** tannic acid, biocompatibility, cytotoxicity, DNA, PrestoBlue

## Abstract

In this comprehensive study, we delve into the intricate binding properties of tannic acid (TA) and examine their dual role in the realm of biomaterial development. While TA’s properties can enhance the functionality and performance of biomaterials, they also raise concerns regarding potential biases in in vitro biocompatibility assessments. We focus on the relevance and constraints of several widely employed cell viability assays, namely the DNA-based PicoGreen assay, the PrestoBlue assay, and the Live/Dead staining technique utilizing fluorescein diacetate (FDA) and propidium iodide (PI). We investigate how these assays perform when applied to TA-coated scaffolds and cell sheets. Through a detailed presentation of our experimental findings, we juxtapose them through a critical review of the existing literature, allowing us to identify and elucidate the limitations these assays face when assessing TA-based biomaterials. In doing so, we aim not only to enhance the understanding of these potential assay biases but also to provide actionable recommendations for accurately evaluating the biocompatibility of TA-modified substances. This dual approach, combining empirical research with literature analysis, offers vital insights for the research community, ensuring that the assessment of TA-coated biomaterials is scientifically sound and reproducible.

## 1. Introduction

Tannic acid (TA) is a high-molecular-weight, water-soluble polyphenol widely used across various industries, including food, animal health, textiles, and medicine, due to its antimicrobial activity, free-radical-scavenging ability, and strong complexing properties [1,2,3,4]. In recent years, its biomedical applications have expanded significantly, leading to the development of TA-based biomaterials for wound dressings with antimicrobial and anti-inflammatory properties, medical adhesives with high bonding strength, implant coatings with antibacterial properties, and controlled drug delivery systems [5,6,7]. As a result, more than 12,000 scientific articles involving the use of TA have been published over the last decade, many of which focus on its antibacterial properties and use in biomedical coatings.

The widespread use of TA in biomaterial science is primarily attributed to its unique chemical structure, which features multiple catechol and galloyl groups that serve as strong hydrogen-bond donors. These functional groups facilitate robust hydrogen bonding, hydrophobic interactions, and electrostatic interactions with various biomolecules, including proteins, polymers, enzymes, and nucleic acids [8]. While these properties have enabled the development of highly functional and stable biomaterials, little attention has been paid to TA’s high reactivity potential, which can interfere with standard characterization assays. By binding to key molecules involved in detection, quantification, and labeling, such as DNA, fluorescent dyes, and proteins, TA can introduce biases that compromise assay accuracy. To the best of our knowledge, most of the current literature does not account for these possible interferences, raising concerns about the reliability of data in in vitro biocompatibility assays.

Many commonly used biocompatibility assays rely on fluorescence- or absorbance-based reagents to assess cell viability, proliferation, or metabolic activity. However, TA’s strong binding affinity for key assay components, such as DNA in quantification assays or fluorescent dyes in Live/Dead imaging, can lead to inaccurate or misleading results. While TA-DNA interactions have been extensively studied and utilized in designing mechanically stable hydrogels and drug delivery systems [9,10], their potential to interfere with DNA detection and quantification remains largely overlooked. Studies on plant extracts rich in polyphenols, including TA, have reported challenges in DNA extraction, polymerase chain reaction (PCR), and loop-mediated isothermal amplification (LAMP) due to strong polyphenol–DNA complexation [11,12,13]. However, to the best of our knowledge, no studies have systematically investigated the impact of TA on DNA-based cytotoxicity assays in the context of biomaterials.

Beyond DNA quantification, TA’s strong DNA-binding affinity can also disrupt assays that rely on DNA-binding dyes, such as those used in Live/Dead staining. For example, TA significantly reduces the emission intensity of ethidium bromide. This dye stains dead cells by intercalating into DNA, which becomes accessible when the cell membrane is compromised [14].

In addition to interfering with nucleic acid binding, detection, and quantification, TA can quench the fluorescence of proteins, which exhibit intrinsic fluorescence due to aromatic amino acids such as phenylalanine, tyrosine, and tryptophan [15]. Consequently, TA may interfere with protein quantification assays by binding to proteins, quenching their fluorescence, and making them unavailable for detection, thereby affecting both fluorescence-based methods and colorimetric assays such as the Bradford and bicinchoninic acid (BCA) assays.

Furthermore, TA can interfere with enzyme-based assays. For instance, in MTT biocompatibility assays, TA can directly reduce tetrazolium salts to formazan without the involvement of living cells, leading to the overestimation of cell viability [16]. Similarly, TA can interfere with laccase-detection assays, which measure the activity of laccase, a copper-containing enzyme involved in phenolic compound oxidation. By chemically reducing assay substrates such as 2,2′-azinobis(3-ethylbenzthiazoline-6-sulfonic acid) (ABTS), TA can decrease detectable laccase activity, resulting in inaccurate measurements [17].

While significant efforts have been dedicated to improving the bioactivity and functionality of TA-based materials, a critical question remains: *How reliable are standard biocompatibility assays when evaluating these materials? Could TA interactions introduce false-negative results, leading to the underestimation of cytotoxic effects?* If TA interferes with assay components, it could obscure toxic responses and misrepresent the true cytocompatibility of TA-based biomaterials.

**Figure 1:**
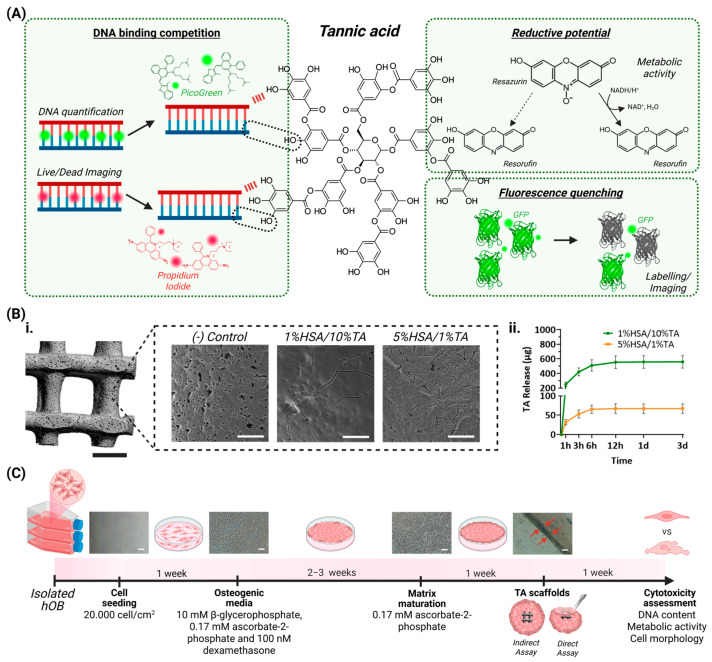
A schematic representation of the study design. (**A**) Tannic acid (TA) can interfere with biocompatibility assays by binding DNA via hydrophobic interactions and hydrogen bonding, non-specifically reducing compounds such as resazurin, and by quenching the fluorescence of proteins and fluorophores. (**B**) The 3D-printed mPCL scaffolds used to evaluate TA assay interference. (**i**) Scaffolds including uncoated controls and scaffolds coated with 1% or 10% TA. Scale bars: scaffold = 1 mm; insets = 50 µm. (**ii**) The TA release profiles from coated scaffolds [7]. (**C**) Biocompatibility testing using cell sheets. A timeline is shown of cell sheet maturation and scaffold testing, including an indirect assay, where TA-coated scaffolds are not in direct contact with cell sheets and TA diffuses into the culture medium, and a direct assay, where cell sheets are wrapped around TA-coated scaffolds, leading to direct cell–TA interaction.

In this study, we highlight how the unique binding properties of TA, while beneficial for innovative biomaterial development, may introduce unintended biases in in vitro biocompatibility assessments. Specifically, we evaluate the applicability and limitations of widely used cell viability assays, including the DNA-based PicoGreen assay, PrestoBlue, and Live/Dead staining using fluorescein diacetate (FDA) and propidium iodide (PI), in the context of TA-coated scaffolds and cell sheets (Figure 1). By presenting our experimental data alongside a critical literature review, we aim to better understand potential assay limitations and offer recommendations for accurately studying TA-based biomaterials.

## 2. Materials and Methods

### 2.1. Materials

All chemicals were purchased from Merck (Darmstadt, Germany) unless otherwise specified.

### 2.2. Methods

#### 2.2.1. Scaffold Fabrication

Macroporous scaffolds made from medical-grade PCL combined with 45% (*w*/*w*) sugar particles, which have crystal sizes ranging from 20 to 50 µm, were fabricated using a 3D printer, BioScaffolder 3.1 (GeSiM mbH, Radeberg, Germany) under the following parameters: an extrusion pressure of 600 kpa, a printing speed of 1.5 mm/s, and a printing temperature of 140 °C. A 0.7 mm nozzle was used to print the scaffolds in a lattice structure, featuring layer-by-layer deposition designed to achieve pore sizes of 1 mm. The printed scaffolds were immersed in ultrapurified water (Arium^®^ pro UF Ultrapure Water System, Goettingen, Germany) for 15 days to leach out the sugar particles and create microporosity on the surface and within the scaffold struts. The fabricated scaffolds were plasma-treated using a vacuum plasma cleaner (PDC-002-HP Harrick Plasma, Ithaca, NY, USA) under O2/Ar (3:1) for 4 min at medium power (38 W) and subsequently sterilized by exposure to 70% ethanol (*v*/*v*) followed by evaporation. The scaffolds were then incubated overnight in 1% HSA and 5% HSA solutions at room temperature with agitation. The resulting layers of 1% HSA and 5% HSA were subsequently stabilized/crosslinked by incubating the HSA-coated scaffolds with 10% TA and 1% TA, respectively, as previously described [7,18].

#### 2.2.2. In Vitro Biocompatibility Study

Human preosteoblasts (hOB) were isolated by explant culture from a male patient undergoing hip arthroplasty, following written informed consent (approved by the QUT Human Research Ethics Committee, approval number 1400001024). Primary hOB cell sheets were formed following protocols previously established by our group [19,20]. Briefly, cells were seeded at a density of 20,000 cells/cm^2^ in 6-well plates and cultured with alpha MEM supplemented with 10% fetal bovine serum (FBS) and 1% penicillin/streptomycin (p/s). Once the cell layers were confluent, the culture medium was supplemented with osteogenic factors (10 mM β-glycerophosphate, 0.17 mM ascorbate-2-phosphate, and 100 nM dexamethasone) to promote cell differentiation and matrix deposition. After 2–3 weeks, the cell sheets were cultured for an additional 7 days in medium supplemented with 0.17 mM ascorbate-2-phosphate to achieve mechanically stable cell sheets easily peeled off the wells as one entity. In the indirect assay, uncoated and coated scaffolds were placed in different wells with the cell sheets. In the direct assay, cell sheet constructs were detached from the wells and wrapped around the scaffolds. In both cases, the cell sheets were cultured for an additional 7 days in medium without osteogenic factors. Afterward, the cell sheets were collected for DNA quantification, as well as confocal and scanning electron microscopy imaging.

#### 2.2.3. PrestoBlue Metabolic Assay

The metabolic activity of cell sheets in indirect and direct contact with the scaffolds was quantified via a PrestoBlue (Thermofisher Scientific, Brisbane, Australia) assay. At day 3 and day 7, the culture medium was aspirated, and 500 µL of medium containing 10% PrestoBlue (%*v*/*v*) was added and incubated for 1 h; fluorescence was measured with a plate reader (BMG LABTECH, Ortenberg, Germany) at wavelengths of 560 nm and 595 nm, together with blank controls using medium with PrestoBlue only.

#### 2.2.4. PicoGreen Assay

After 7 days in culture, the cell sheets were rinsed with PBS and stored at −20 °C until analysis (average of n = 3 biological replicates). The samples were incubated in proteinase K (Thermofisher Scientific, Australia) at 0.5 mg/mL in phosphate-buffered EDTA (pH 7.1) at 37 °C on a shaker overnight to obtain the lysate. The following day, the samples were incubated at 56 °C for 8 h and centrifuged at 2000 RPM for 5 min to remove debris and undigested ECM. Double-stranded DNA content was quantified in the lysate supernatant using a Quant-iT™ PicoGreen^®^ dsDNA quantification assay (Thermofisher Scientific, Australia) following the manufacturer’s instructions.

#### 2.2.5. Statistical Analysis

A minimum of four experimental replicates (unless otherwise mentioned) were used in each study, and the results are presented as the mean value ± standard deviation. The effect of HSA/TA surface treatment in each assay compared to the controls was analyzed using two-way ANOVA (GraphPad Prism 9 software, USA). Differences between the groups were analyzed using the Tukey test of multiple comparisons, and a confidence level of *p* < 0.05 was considered to be statistically significant, unless otherwise specified.

## 3. Results and Discussion

While TA is a highly versatile molecule with numerous biomedical applications, no studies, to the best of our knowledge, have reported its potential interference with standard in vitro characterization assays. This study aims to determine whether the cytotoxic effects observed in TA-containing biomaterials reflect an accurate biological response or an artifact of assay interference.

**Figure 2:**
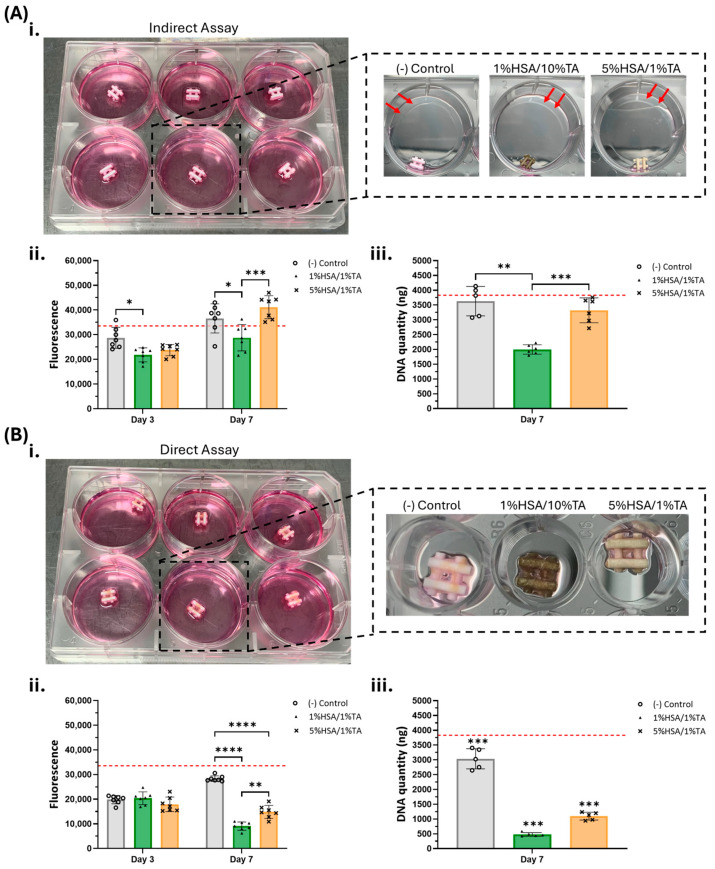
Biocompatibility assessment of uncoated and TA-coated scaffolds. Experimental approaches exposing TA-coated scaffolds to mature cell sheets: (**A**) Indirect assay: Cell sheets remained attached to the bottom of the well, exposing them to TA released from the scaffold in a diluted form, minimizing potential accumulation and toxicity. Red arrows indicate cell sheets attached to the well surface (**i**). (**B**) Direct assay: Cell sheets were wrapped around the coated scaffolds, allowing for direct cell–TA interactions and the potential accumulation of TA within the cell sheet. Biocompatibility was assessed quantitatively by measuring metabolic activity via a PrestoBlue assay at 3 and 7 days in culture (**ii**), and the DNA content after 7 days in culture (**iii**); red dotted lines represent the metabolic activity and DNA content of cell sheets at day 0, prior to the assays. All measurements are reported as the average ± standard deviation (SD). * *p* < 0.05; ** *p* < 0.01; *** *p* < 0.001; **** *p* < 0.0001, (n = 6).

To address this, we designed two experimental approaches applying TA-coated scaffolds to mature cell sheets. The use of cell sheets not only enables controlled and targeted cell delivery, as they form dense, homogeneous layers, but also provides a highly organized cellular environment embedded in their self-synthesized extracellular matrix (ECM), creating a physiologically relevant, tissue-like structure for biomaterial cytotoxicity testing. In the indirect assay, cell sheets remained attached to the bottom of the well, exposing them to TA released from the scaffold in a diluted form, minimizing potential accumulation and toxicity (Figure 2A(i)). In contrast, cell sheets were wrapped around the coated scaffolds in the direct assay, leading to direct cell–TA interactions and allowing TA to accumulate within the cell sheet, potentially being released during sample processing (Figure 2B(i)).

We evaluated the interference of TA with widely used cell viability and proliferation assays, including the DNA-based PicoGreen assay, PrestoBlue, and Live/Dead staining with fluorescein diacetate (FDA) and propidium iodide (PI). Additionally, to assess the concentration-dependent effects of TA on assay performance, we tested scaffolds coated with either 1% or 10% TA, both of which have been previously characterized and applied as antibacterial coatings for medical-grade polycaprolactone scaffolds in tissue engineering applications [7,18].

By comparing these conditions—alongside controls using TA-free scaffolds—we aimed to distinguish between actual cytotoxicity and assay-related artifacts. Our findings highlight the need to critically evaluate biocompatibility assay results when testing TA-containing biomaterials, as the potential for false-positive cytotoxicity generally remains overlooked in this field.

Figure 2 presents the DNA quantification results from the PicoGreen assay and the metabolic activity of cell sheets exposed to TA-coated scaffolds through both direct and indirect approaches. In the indirect assay, metabolic activity remained largely unchanged across groups after three days in culture. However, a slight decline was observed at day 7 in the group exposed to the highest TA concentration (1%HSA/10%TA). In contrast, DNA quantification revealed no substantial differences between the controls and the lower TA concentration (5%HSA/1%TA). Still, a decrease was observed in the 1%HSA/10%TA group, suggesting a cytotoxic effect at higher TA concentrations. Nonetheless, Live/Dead staining showed no significant differences among groups in terms of live cell populations and cell morphology (Supplementary Figure S1A).

Similarly, in the direct assay, metabolic activity remained comparable across all groups at day 3. However, by day 7, both metabolic activity and DNA content had significantly declined in the TA-coated groups compared to the controls, with a more pronounced effect at the highest TA concentration, indicating a dose-dependent response. This decrease was also evident in the lower fluorescence signal of FDA-stained live cells (Supplementary Figure S1B) in the TA-coated groups relative to the controls.

Despite this, the scanning electron microscopy (SEM) images of cell sheets wrapped around both uncoated and TA-coated scaffolds showed that all scaffolds were fully covered with densely packed cell sheets with substantial amounts of secreted mineralized matrix (Supplementary Figure S2A(i–iii)). Further analysis of cell morphology and distribution by confocal microscopy, where actin filaments were stained with phalloidin and nuclei with DAPI (Supplementary Figure S2A(iv,v)), confirmed highly dense, elongated, and well-organized fiber alignment. This indicated comparable cell organization and density across all groups, suggesting no visible cytotoxic effects from TA.

These morphological findings suggest that despite reductions in DNA content and metabolic activity, the cell sheets remained structurally intact, with no signs of disruption or detachment. This discrepancy between quantitative and qualitative biocompatibility assessments raises the question of whether TA may interfere with the assays used in this study. Given TA’s high reactivity and strong binding affinity to biomolecules such as nucleic acids, it is plausible that it could affect the accuracy of specific viability assays.

For instance, cell proliferation is commonly assessed by quantifying DNA content using DNA-binding fluorophores such as CyQuant, PicoGreen, and SYBR Green I. Specifically, PicoGreen binds to double-stranded DNA (dsDNA), increasing its fluorescence signal by over 1000-fold upon binding, allowing for the highly sensitive detection and quantification of as few as 100 cells [21]. However, the accuracy of the PicoGreen assay can be influenced by the presence of interfering compounds. For instance, Koba et al. reported that DNA-intercalating drugs such as mitomycin C, mitoxantrone, actinomycin D, and doxorubicin alter the fluorescence intensity of dsDNA-PicoGreen complexes by competing for DNA-binding sites [22]. Given TA’s strong affinity for DNA, it is possible that TA present in the culture medium or accumulated in the cell sheets matrices similarly interferes with PicoGreen’s ability to bind dsDNA, thereby skewing fluorescence-based DNA quantification.

Interestingly, the presence of polyphenols has been reported to be a major challenge in DNA extraction from polyphenolic-rich materials such as brown algae and green tea [23,24]. For instance, Snirc et al. reported that polyphenolic compounds negatively impact DNA yield from brown algae when using Qiagen/PicoGreen assays. Although the authors optimized the extraction and purification process by incorporating an additional chloroform/isoamyl alcohol purification step to enhance DNA purity, the yield remained significantly reduced in the presence of polyphenols [24]. Similarly, TA has been shown to interfere with DNA quantification by real-time PCR due to free phenolic groups, which oxidize into quinones and covalently bind to DNA polymerase, leading to its inactivation. Additionally, TA also reduced DNA availability in the sample [12,13]. More recently, Nwe et al. reported that TA inhibits loop-mediated isothermal amplification (LAMP), a technique used for direct DNA amplification and detection, in a dose-dependent manner. The authors demonstrated that TA reduced the amplicon signal, with complete inhibition observed at concentrations exceeding 100 ng/µL [11]. Finally, Liu et al. showed that nanoparticles loaded with Zn and TA could bind to PicoGreen-bound DNA, decreasing fluorescence signals [25].

Taken together, these findings suggest that our study’s observed reductions in DNA content may not necessarily reflect true cytotoxicity but, rather, an artifact of TA interference with the PicoGreen assay. Furthermore, the differences in DNA quantification between the indirect and direct assays suggest that TA accumulation within the cell sheet matrix may further impair assay performance. TA released during sample digestion before DNA quantification could act as a confounding factor—not only through direct optical interference but also by potentially binding to dsDNA. A clear indication of this interference is the persistent TA-dependent color change in the solution after digestion and DNA extraction (Supplementary Figure S3). Solutions containing extracted DNA from samples with higher TA concentrations appear darker, likely due to the oxidation of TA released from the scaffolds. This effect is more pronounced in the direct assay, where cell sheets were wrapped around the scaffolds (Supplementary Figure S3B), compared to the indirect assay, where a color change is observed but is less intense. Notably, this discoloration is also visible on the scaffolds (Figure 2), even after being covered with cell sheets.

Importantly, this concern extends to other fluorescence-based DNA quantification assays commonly used to evaluate biomaterial biocompatibility, particularly in studies involving TA-based materials, where potential assay biases are often overlooked. It also applies to cell viability assays that rely not on DNA quantification but on the enzymatic activity of metabolic enzymes secreted by viable cells. For instance, studies have shown that phenolic compounds in green tea and *Terminalia ferdinandiana* (a native Australian fruit) can interfere with tetrazolium-based assays like MTT and MTS (3-(4,5-dimethylthiazol-2-yl)-5-(3-carboxymethoxyphenyl)-2-(4-sulfophenyl)-2H-tetrazolium)-based assays [16,26]. These assays rely on the reduction of tetrazolium compounds to formazan by metabolically active cells. However, phenolic antioxidants can directly reduce tetrazolium compounds, leading to the overestimation of cell viability. While mitochondrial dehydrogenases primarily reduce MTT via NADH, MTS reduction involves NADPH/NADH and extracellular reducing agents, such as glutathione. This effect is likely due to both increased dehydrogenase activity in treated cells and the intrinsic reductive potential of the extracts [26]. While there is limited direct evidence of interference by TA or other polyphenols on metabolic assays like resazurin-based assays (e.g., PrestoBlue, AlamarBlue), which rely on the reduction of resazurin to the fluorescent molecule resorufin by metabolically active cells, the reductive capacity of polyphenols suggests that they could non-specifically reduce resazurin, leading to false signals. Therefore, when assessing cell viability in the presence of TA or similar compounds, it is advisable to include appropriate controls to account for potential assay interference. Overall, cell viability assays based on redox reactions may not be suitable for testing antioxidant/polyphenol-rich samples and could lead to biases and false positives when evaluating the biocompatibility of these materials.

Table 1 summarizes the most commonly used assays for evaluating biomaterial biocompatibility, emphasizing their underlying principles, their known limitations, and the potential for interference by TA. Due to its strong DNA-binding affinity, reductive potential, and fluorescence quenching properties, TA can significantly alter the outcomes of assays that rely on fluorescence, enzymatic reduction, or membrane integrity. These interferences should be carefully considered when designing and interpreting biocompatibility studies involving TA-modified materials. This brief report aims to raise awareness of these potential interferences—an issue that, to the best of our knowledge, has not been sufficiently acknowledged in the literature. However, further studies are necessary to better understand the mechanisms involved. Future work should incorporate comprehensive analyses of the nature and stability of TA-assay reagent interactions using complementary chemical characterization techniques. Additionally, investigations into TA’s effects when it is bound to biomaterials, released into the surrounding medium, or retained within hydrogel-crosslinked systems should be conducted.

## 4. Conclusions

TA’s interactions with DNA and its potential interfere with various molecular and cell-based assay methods highlight significant biases in evaluating the in vitro biocompatibility of TA-based materials. These interactions, including DNA-binding, fluorescence quenching, color changes due to TA oxidation, and the direct reduction of tetrazolium compounds in metabolic assays, can lead to inaccurate cell viability readings and erroneous results. Despite the exponentially growing body of research on TA and other polyphenolic compounds, few studies acknowledge these potential assay interferences, and they are rarely considered when interpreting data. This oversight underscores the importance of recognizing and addressing these biases in experimental design. To more accurately assess the biocompatibility of TA-based materials, it is essential to use a combination of quantitative and qualitative assays, including those that do not rely on redox reactions or DNA quantification, to provide a scientifically accurate and reproducible study. By implementing appropriate controls and utilizing a broader range of assays, researchers can better understand the interactions between TA and biological systems, ensuring the reliability of findings and the safe development of TA-based biomaterials.

## Figures and Tables

**Table 1:** Overview of commonly used biocompatibility assays and their principles, limitations, and potential interactions with TA [14,21,22,27,28,29,30,31].

Assay	Principle	Limitations	Possible Interactions with TA	Key References
**PicoGreen**	Fluorescent dye selectively binds to double-stranded DNA; fluorescence correlates with DNA content. Limit of detection: 25 pg/mL ds DNA, ~100 cells.	Fluorescence intensity can be affected by salts or other compounds; quenching and binding interference may alter results.	TA binds DNA and may block dye binding or quench fluorescence, reducing signal.	[21,22]
**CyQuant**	Fluorescent dye binds DNA; fluorescence proportional to cell number. Limit of detection: 10–50 cells.	Quenching agents can interfere; CyQUANT binds all DNA and cannot distinguish between live, dead, and apoptotic cells.	TA may bind DNA and/or quench CyQuant fluorescence.	[27]
**Alamar Blue/ PrestoBlue**	Resazurin is reduced to fluorescent resorufin by metabolically active cells.	Non-specific reduction by redox-active compounds and interference from test compounds can affect resorufin fluorescence, depending on assay conditions.	TA may reduce resazurin, causing false viability results, and interfere with fluorescence by quenching or obscuring the solution.	[28]
**MTT**	Yellow MTT salt is reduced to purple formazan by mitochondrial enzymes in viable cells.	Results can be influenced by factors such as cell density, medium composition, and the presence of chemicals or nanoparticles that non-specifically reduce MTT salts. Insoluble formazan can also damage cell structure.	TA’s reductive properties may reduce MTT directly, generating false-positive signals.	[16,29]
**LDH**	Measures lactate dehydrogenase released from damaged cells into the medium.	Lack of specificity, as LDH is released not only during cell death but also in response to cell stress or injury. The assay can be affected by interfering compounds that influence LDH activity or absorption readings, leading to false positives.	TA may inhibit or denature LDH enzyme activity, affecting signal strength.	[30]
**Live/Dead imaging**	FDA: Membrane-permeable dye hydrolyzed by esterases in viable cells to produce green-fluorescent fluorescein. PI: Membrane-impermeable dye that stains DNA of dead cells, emitting red fluorescence. Other dyes: Calcein-AM, SYTOX Green, Hoechst, EthD-1.	Fluorescence quenching; presence of other DNA-binding molecules can interfere with the specific labeling of dead cells; qualitative.	TA may quench fluorophores or interfere with DNA-binding dyes such as PI or EthD-1.	[14,31]

## Data Availability

The raw data supporting the conclusions of this article will be made available by the authors on request.

## Supplementary Materials

The following supporting information can be downloaded at: https://www.mdpi.com/article/10.3390/bioengineering12060660/s1, Figure S1: Live/Dead staining of cell sheets exposed to control and TA-coated scaffolds via (A) Indirect and (B) Direct assays after 7 days; Figure S2: Morphological analysis of cell sheets wrapped on (A) control, (B) 1%HSA/10%TA- and (C) 5%HSA/1%TA-coated scaffolds after 7 days in culture. Figure S3: TA-dependent color change after digestion and DNA extraction in (A) indirect and (B) direct assay samples.
