# Supplementary material for: Assessing the Biocompatibility of Tannic Acid-Based Biomaterials: Addressing Challenges in Standard Cytotoxic Assays
Source: Bioengineering (Basel). 2025 Jun 16;12(6):660. doi: 10.3390/bioengineering12060660 (PMC12189109; doi:10.3390/bioengineering12060660)
Supplement: Supplementary file 1 [file bioengineering-12-00660-s001.zip › bioengineering-3668318-supplementary.pdf]

Assessing the Biocompatibility of Tannic Acid-Based Biomaterials: Addressing Challenges in Standard Cytotoxic Assays

Silvia Cometta [1,2,3,*] and Dietmar Werner Hutmacher [1,2,3,4,*]

[1] Faculty of Engineering, School of Mechanical, Medical and Process Engineering, Queensland University of Technology, Brisbane, QLD 4000, Australia
[2] Australian Research Council Training Centre for Multiscale 3D Imaging, Modelling and Manufacturing (M3D Innovation), Queensland University of Technology, Kelvin Grove, QLD 4059, Australia
[3] Max Planck Queensland Centre, Queensland University of Technology, Brisbane, QLD 4000, Australia
[4] Australian Research Council Training Centre for Cell and Tissue Engineering Technologies, Queensland University of Technology, Brisbane, QLD 4059, Australia
* Correspondence: silvia.comettaconde@qut.edu.au (S.C.); dietmar.hutmacher@qut.edu.au (D.W.H.)

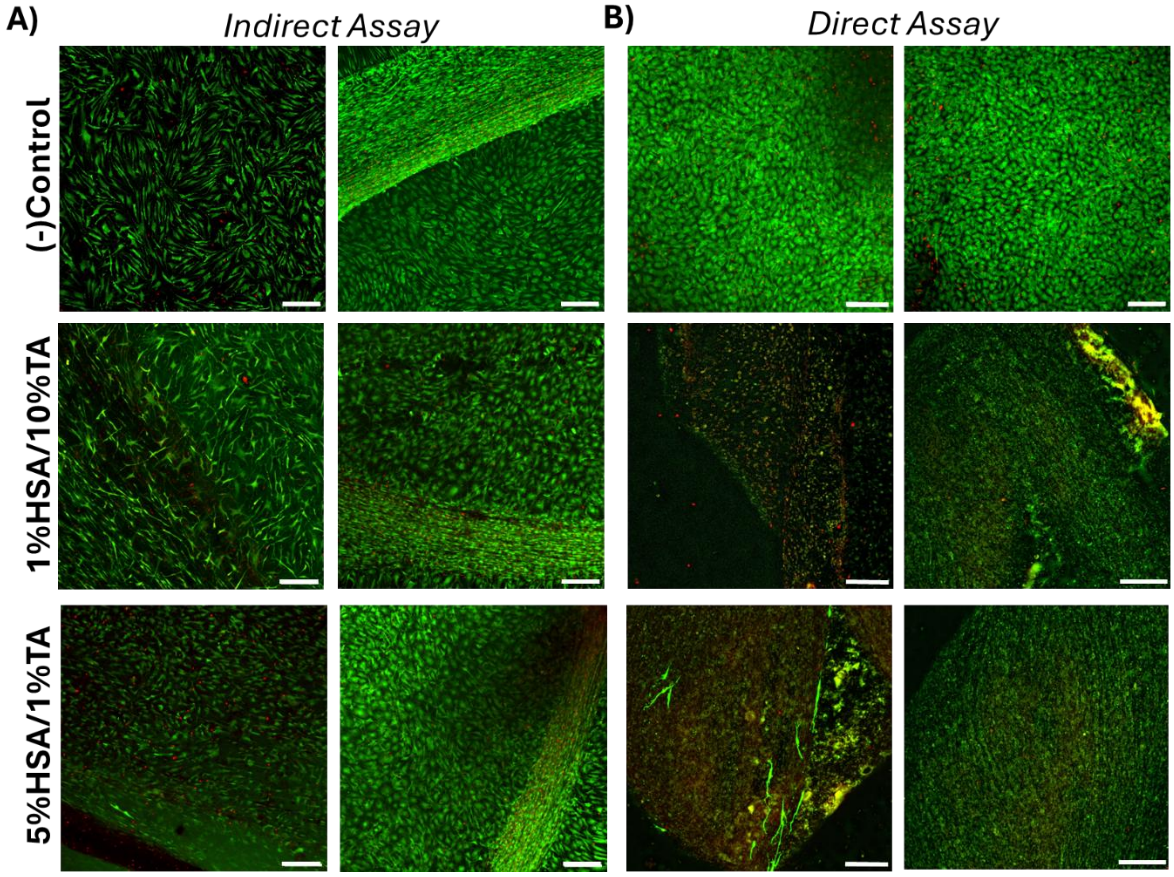

Figure S1. Live/Dead staining of cell sheets exposed to control and TA-coated scaffolds via A) Indirect and B) Direct assays after 7 days. No significant differences between groups regarding live cell populations and cell morphology were observed in the indirect assay. However, cell sheets wrapped around TA-coated scaffolds (direct assay) exhibited a significantly lower fluorescence signal of FDA-stained live cells than the control group. Scale bars: 200 µm

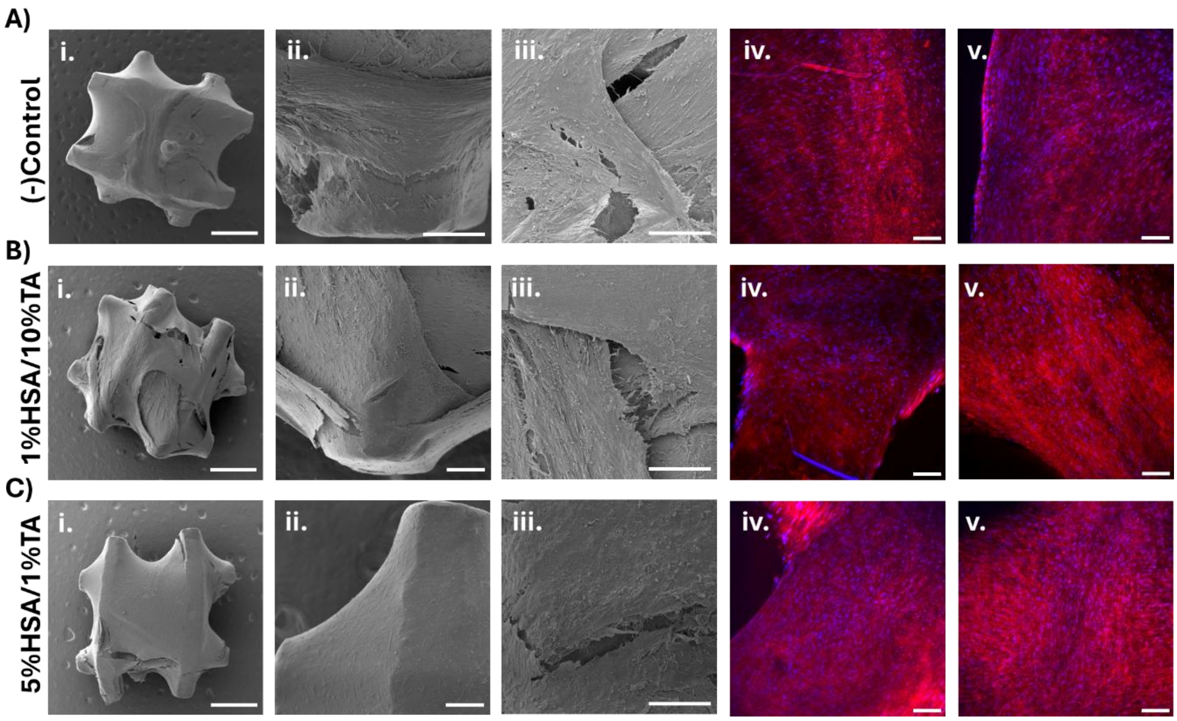

Figure S2. Morphological analysis of cell sheets wrapped on A) control, B) 1%HSA/10%TA- and C) 5%HSA/1%TA-coated scaffolds after 7 days in culture. (i-iii) SEM images showing that all scaffolds were fully covered by densely packed cell sheets with substantial amounts of secreted mineralized matrix. (iv-v) confocal microscopy of samples stained for nuclei (blue, DAPI) and actin filaments (red, phalloidin) confirming the presence of highly dense, elongated, and well-organized fibre alignment. Scale bars (i) 1 mm, (ii-ii) 200 µm, (iv-v) 100 µm

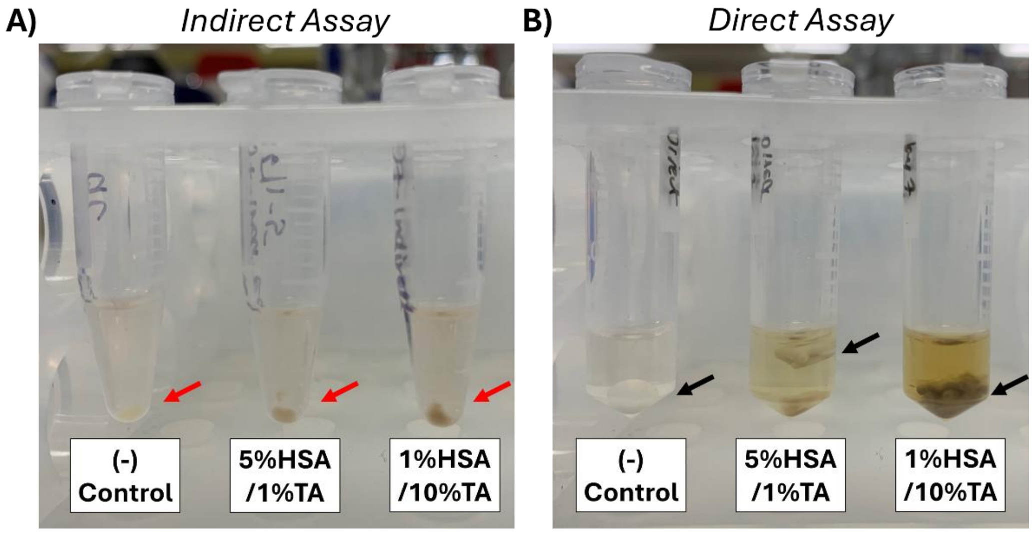

Figure S3. TA-dependent color change after digestion and DNA extraction in A) indirect and B) direct assay samples. Solutions containing extracted DNA from samples with higher TA concentrations appear darker, likely due to the oxidation of TA released from the scaffolds. Red arrows indicate cell sheets, and black arrows indicate cell sheets wrapped around scaffolds after the digestion process.
